# Fracture Resistance of Endodontically Treated Maxillary Premolar Teeth Restored with Wallpapering Technique: A Comparative In Vitro Study

**DOI:** 10.1155/2023/6159338

**Published:** 2023-04-25

**Authors:** Shahed Wissam Abdulamir, Manhal A. Majeed

**Affiliations:** Aesthetic and Restorative Dentistry Department, Collage of Dentistry, University of Baghdad, Baghdad, Iraq

## Abstract

**Objectives:**

This in vitro study aimed to evaluate and compare the fracture resistance and mode of failure of endodontically treated maxillary premolar teeth restored with different direct composite restorative techniques.

**Materials and Methods:**

Forty freshly extracted maxillary premolar teeth with comparable sizes were used in this in vitro study. Each tooth received mesio-occluso-distal cavity preparation (3 mm width and 6 mm depth) followed by endodontic treatment. Canals were instrumented with RACE EVO rotary files (FKG, Dentaire, Switzerland) up to MAF 25/.06. Canals were obturated using a single cone technique, then the teeth were divided arbitrarily into five groups (*n* = 8): *Group A*: direct composite resin only using a centripetal technique, *Group B*: direct composite resin with glass fiber post, *Group C*: direct composite resin with short fiber-reinforced composite (everX Flow), *Group D*: direct composite resin with leno wave ultra high molecular weight polyethylene (LWUHMWPE) fibers placed on cavity floor, and *Group E*: direct composite resin with LWUHMWPE fibers placed circumferentially around the cavity walls (wallpapering technique). The teeth were then stored in distilled water at 37°C for 24 hr. The fracture resistance of each sample was measured using a universal testing machine in Newton (N). The data were analyzed using one-way analysis of variance (ANOVA) and the Bonferroni test with a significance level of 0.05.

**Results:**

Group E recorded the maximum mean of fracture load (2,139.375 N), while Group A recorded the minimum mean of fracture load (689.6250 N). The one-way ANOVA test revealed a significant difference between the groups. The Bonferroni test showed a significant difference between each two groups, with the exception of those between Groups B and C and between Groups D and E, where there were no statistically significant differences (*p* > 0.05).

**Conclusion:**

Restoration of endodontically treated teeth using the wallpapering technique recorded the highest mean of fracture resistance with a repairable mode of fracture.

## 1. Introduction

Restoration of nonvital premolars with mesio-occluso-distal (MOD) cavities is still a challenging procedure due to the extensive amount of tooth structure lost from caries, old fillings, and/or endodontic treatment, which causes weakening of these teeth [1]. In terms of fracture resistance, MOD cavity preparation of these teeth represents the worst scenario [2]. Access cavity preparation decreases the toughness of premolars by only 5%, while MOD cavity preparation decreases tooth toughness by 14%–44% [3].

In restorative dentistry, composite resin materials are, in general, the first options in anterior [4, 5] and posterior restorations [6] due to many advantages like excellent esthetic, conservation of tooth structure, and economic restorative material [6], on the other hand, composite restorations have a failure rate of around 5% based on wear and fractures [7, 8]. In terms of fracture resistance, resin composite can provide some strengthening effect; however, this positive effect is material dependent and must be considered carefully when selecting a suitable composite material [9]. However, endodontically treated teeth (ETT) with significant loss of coronal tooth structure may not provide adequate support for restoration [10, 11], and thus catastrophic failure may occur due to the lower proportion of the residual dentino-enamal complex region and the lack of toughness of composite materials [12]. In such teeth, a prefabricated glass fiber post may be joined with composite resin restorations [10, 13]. Glass fiber posts are identical to dentin in terms of modulus of elasticity and can effectively distribute forces down the long axis of the post when subjected to compressive loads, decreasing the opportunity of root dentin fracture [14]. However, these posts have certain drawbacks, as prefabricated posts require additional root canal preparation, resulting in dentin loss and making the root more susceptible to fracture [15].

Nowadays, numerous types of fibers with different formulations and compositions are commercially available with the aim to reinforce composite restoration and increase mechanical properties. One of the fiber formulations introduced is short fiber-reinforced composite (SFRC) which claimed to provide reinforcement in three dimensions [16]. The material is planned to restore vital and non-vital teeth as a bulk base in high-stress-bearing areas.

Another alternative reinforcement adhesive material is the leno wave ultra high molecular weight polyethylene (LWUHMWPE) fiber. The construction of the fibers is based on locked nodal intersections and multidirectional yarns that create a great multitude of load paths that redistribute the occlusal forces through a greater region of dental restoration [17]. These fibers are significantly more resistant to breaking than fiberglass and must be cut using specially designed scissors; having virtually no memory, the open and lace-like framework of the leno woven ribbon provides it with the ability to conform closely to the contours of the teeth and dental arch [18]. Because of the leno weave or triaxial braid, the material has a three-dimensional structure, and it can be used in both fixed and removable prosthodontics, space maintainers, endodontic posts and cores, and splinting in dentistry [19, 20]. The manufacturers of polyethylene ribbond fibers are made of aligned polymer chains with a low modulus of density, allowing for greater impact resistance [21].

A vital characteristic of any restorative material is fractural resistance. It shows a material's ability to withstand fracture and cracking [22]. Fiber reinforcement of composite resin materials improves the material's fracture resistance and flexural strength [23, 24].

Despite the wide versatility of the available reinforcing materials, there is not enough scientific evidence to help the clinician suggest which material to select. Therefore, the objective of this study was to evaluate the effect of using different fiber formulations (glass fiber post, SFRC, and LWUHMWPE) when used in combination with direct composite for the restoration of root-filled maxillary premolar teeth in vitro.

## 2. Materials and Methods

### 2.1. Sample Selection

This research was approved by the Research Ethics Committee of the College of Dentistry, University of Baghdad, Iraq, in January 2022 (no. 469522). For this in vitro experiment, 40 freshly extracted, sound human maxillary premolars with two roots were collected from several dental clinics. Only sound teeth with no caries, restoration, or cracks as examined by trans-illumination and with normal occlusal anatomy and of comparable size were included [25]. The storage solution for the disinfected teeth was distilled water. To minimize confounding variables, the maximum buccolingual dimension, mesiodistal dimension, and occlusogingival height of each tooth were determined using a digital caliper [26].

To ensure uniformity of tooth size within each group, the aforementioned dimensions of each tooth within a group should not vary more than 10% from the measured means of these dimensions for that group [27]. To establish uniform tooth size among the five groups, a one-way analysis of variance (ANOVA) test was performed for each of the dimensions mentioned earlier, which revealed no statistically significant differences (*p* > 0.05).

### 2.2. Sample Preparation

Prior to cavity preparation, a flowable composite (Tetric N-Flow, Ivoclar Vivadent, Liechtenstein) was employed to take an imprint for the occlusal surface of each tooth to minimize the requirement for finishing and polishing by restoring each tooth back to its original occlusal anatomy [28].

Each tooth then received a standardized class II MOD cavity preparation (3 mm buccolingual width and 6 mm occlusal depth) assessed from the tip of the buccal cusp without a proximal step. For cavity preparation, flat-end diamond fissure bur (no. 121415, Shofu Inc, Japan) in an air turbine handpiece mounted on a modified dental surveyor ensures parallelism between the long axis of the bur and the tooth [29]. To ensure standardization, a periodontal probe and caliper were used to check the cavity depth and dimensions.

### 2.3. Endodontic Treatment

The roof of the pulp chamber was removed using a diamond round bur (no. 2128C, Microdont, Brazil) and an ENDO-Z bur (Dentsply, Maillefer, Switzerland) utilizing a high-speed handpiece mounted in a modified dental surveyor and copious amounts of water cooling. The working length of each tooth was then manually assessed using the #10 K file in the canal until seen from the apical foramen, then subtracting 1.0 mm from the apical foramen to determine the final working length. Teeth were prepared using RACE EVO Ni-Ti rotary system (FKG, Dentaire, Switzerland) with E-connect S Endo Motor (Eighteeth, China). Root canal instrumentation was performed in a crown-down manner using gentle in and out with copious irrigation of 2 ml of 5.25% NaOCl between each two files. The canals were instrumented in the following sequence: RE1, RE2, and RE3, which corresponds to #25/06. Single cone obturation was then done with matched gutta-percha cones (Diadent, Korea) and resin-based sealer (Sealapex, Kerr, USA).

### 2.4. Sample Grouping

According to the restorative technique, the teeth were randomly divided into five groups (*n* = 8) following the previous study [30–33]: *Group A*: teeth restored conventionally with direct composite using the centripetal technique, *Group B*: direct composite with glass fiber post, *Group C*: direct composite with SFRC, *Group D*: direct composite with polyethylene ribbond fibers placed on the cavity floor, and *Group E*: direct composite with polyethylene ribbond fibers adapted circumferentially around the cavity walls (wallpapering technique).

### 2.5. Restorative Procedure

For all groups, the proximal walls (mesial and distal) were built up first using G-aenial A'CHORD universal composite, then the remaining cavity was restored following the sample grouping

The prepared cavity of each tooth was etched with 37% phosphoric acid (Super Etch, SDI, Australia) for 20 s, then rinsed with water for 5 s and air-dried. A micro brush was then used to apply G-Premio BOND universal adhesive (GC Europe, Belgium) to the tooth structure, wait for 10 s, then air dried gently for 5 s and light cured for 20 s using an eighteeth curing pen and placing it as close to the cavity as possible with an intensity of 1,000 mw/cm^2^ (Eighteeth, China) following the manufacturing instructions. SuperMat™ Universal Matrix Tensioning System (0.038 mm thickness and 6.3 mm height) (Kerr, USA) was used in all samples. The universal composite G-aenial A'CHORD (GC Europe, Belgium) was used to build up the proximal walls in all groups with wall a thickness of 1 mm up to the marginal ridge level using a plastic instrument (CONDENSA LM-Arte™), then light cured for the 20 s.

Group A: In this group, the remaining cavity was restored using the horizontal incremental layering technique using the universal G-aenial A'CHORD with layer thickness for 2 mm of each increment. Each layer was light-cured for 20 s using a light-curing unit follow the manufacturing instructions. After the placement of the final layer of composite resin and prior to its curing, Teflon was wrapped on the composite layer, then the prefabricated stamp was placed on the top of this layer to restore the original occlusal anatomy of each tooth, and light cured for 20 s.

Group B: In this group, a glass fiber post was inserted into the palatal canal of each tooth (GC fiber post, GC America). #1 with 0.5 mm apical diameter and 1 mm coronal diameter was used. For postspace preparation, a corresponding post drill mounted on a slow-speed handpiece was used, leaving about 5 mm of the gutta percha apically. The canal was then rinsed with 1 ml of saline and air dried thoroughly using paper points. G-Multi primer (GC Europe, Belgium) was used for treating the post surface prior to its cementation. The self-adhesive resin cement (GC Europe, Belgium) was then injected into the canal, and the post was inserted after 1 min of cement application, following the manufacturing instructions, after that light curing for 20 s, then the material was allowed to be set chemically for 4 min. The remaining cavity was restored using a horizontal layering technique as for Group A.

Group C: In this group, the remaining cavity was restored with SFRC (everX Flow^TM^/bulk shade, GC Europe, Belgium). After the buildup of the proximal walls as for Group A, SFRC was applied in bulk, leaving 2 mm occlusally from the cavosurface margin, and light-cured 20 s follow manufacturer's instructions. The residual cavity was then restored as for Group A.

Group D: In this group, after the buildup of the proximal walls with composite resin as for other groups, a 5 mm piece of ribbond was cut from the 4 mm wide ribbond sheet using ribbond scissor (Ribbond-Ultra; Ribbond Inc., Seattle WA, USA) and inserted in the floor of the cavity in a buccolingual direction. Before its application, the ribbond fiber was dampened with a wetting resin (Ribbond wetting resin, Ribbond Inc., Seattle WA, USA); the excess wetting resin was squeezed out of the ribbon fiber using a plastic instrument. The ribbond piece was then covered with a thin layer of flowable composite (Ribbond Securing composite, Ribbond Inc., Seattle WA, USA) and adapted well to the floor of the cavity using a plastic instrument and light cured for 20 s. The dimensions of the ribboned piece used were determined according to a pilot study.

Group E: In this group, after the buildup of the proximal walls as for other groups, the cavity was restored with wallpapering technique as described by Deliperi et al. [12], whereby two pieces of ribbond fiber, 4 mm wide by 6 mm long, were C-shaped and used to cover the inside of the cavity walls circumferentially. Before insertion, the fibers were wetted with ribbon-wetting resin and covered with a thin layer of flowable composite; then the first piece of ribbond was adapted to the inside of the buccal wall, while the second was inserted against the inside of the palatal wall, overlapping one another at the proximal surface ending 2 mm below the cavosurface margin and folding down onto the axio-pulpal line angle, after that light cured for 20 s. The remaining cavity was then restored as for Group A. The restored samples were stored in distilled water at 37°C in an incubator for 24 hr.

### 2.6. Fracture Test

The fracture resistance of all samples was measured using a computerized universal testing machine (Laryee, China) in Newton using a single load to failure test. Stainless steel indenter with round end was used to provide force to each tooth in the vertical direction at crosshead speed of 0.5 mm/min. A piece of rubber (1 mm thick) was inserted between the indenter and the tooth to prevent distortion caused by direct contact.

Each specimen was then visually examined using magnifying loups (2.5×) to determine the fracture mode and according to Scotti et al. [4], whether it was a restorable fracture (above cemento-enamal junction (CEJ)), or a nonrestorable fracture (below CEJ).

Statistical analysis was done using SPSS version 22. The Shapiro–Wilk test was employed to determine the normality of distribution. At a level of significance of 0.05, and for determining the significance of the mean difference in fracture resistance among the five groups, a one-way ANOVA test was used, Bonferroni test was used for further comparisons among the groups.

## 3. Results

Shapiro–Wilk test was employed to determine the normality of distribution that showed the results are normally distributed. The descriptive statistics for the various groups, including mean values and standard deviation, are mentioned in Table 1. Group E recorded the highest mean of fracture resistance (2,139.375 N), in which the samples were restored with the wallpapering technique, while Group A recorded the lowest mean value (689.625 N), in which the teeth were restored with direct composite alone.

One-way ANOVA test showed a statistically significant difference (*p* < 0.05), as shown in Table 2.

Further comparisons between each two groups using the Bonferroni test revealed a significant difference between each two groups (*p* < 0.05), except between Group B with Group C and between Group D with Group E, where there were no significant differences (*p* > 0.05), as shown in Table 3.

**Figure 1:**
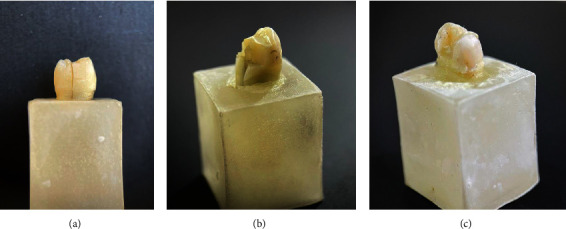
Photographs showing the mode of failure of different groups: (a) Catastrophic failure of Group A, (b) nonrestorable fracture extending below the CEJ of Group B, and (c) restorable fracture of Groups C, D, and E.

Concerning the mode of fracture, most of the samples of Groups C, D, and E showed a restorable fracture, while the majority of the samples of Groups A and B showed a nonrestorable fracture, as shown in Table 4 and Figure 1.

## 4. Discussion

Today, it has been widely accepted that the success of root canal treatment depends not only on the success of root canal treatment but also on the success of the coronal restoration. In daily clinical practice, the restoration of root-filled is a treatment requiring comprehensive restorative planning and can be performed by using indirect restorative techniques or by direct restorative techniques. The main problem with the restoration of root-filled teeth is the reduced elasticity of the tooth and should be addressed when selecting a material for the restoration of these teeth [34].

Direct composite material is the most commonly used for the restoration of such teeth. Composite resin materials do not lack strength, but they do lack toughness. Therefore, this research was conducted with the aim to use different fiber-reinforced materials with different fiber formulations in combination with composite resin in an attempt to increase the fracture resistance of root canal-treated teeth. In posterior teeth, it is quite challenging to reinforce the remaining tooth structure after the endodontic procedure. In terms of relative cusp stiffness, the loss of one marginal ridge results in a mean loss of 46% [35].

Maxillary first premolars were selected for use in this study because their cuspal inclines render them more vulnerable to fracture [36].

Standardized class II MOD cavities were prepared in all samples to stimulate the compromised condition of weakened root canal-treated premolars.

The results of this study revealed statistically significant differences in fracture resistance among the various groups. Group A, in which teeth were restored with direct composite resin alone, recorded the lowest mean value of fracture resistance as compared to the other groups. This could be explained from a mechanical point of view. When an object is subjected to a compression test, as in the single load-to-failure test used in this study, complex stresses within the object will arise, the compression forces are resolved into forces of shear and tensile (Poisson's effect) [37], that are transferred to the cavity walls and floor and may lead to the initiation of a crack. Crack propagation will lead to catastrophic failure due to the inherent lack of toughness of composite resin [38–40]. This is supported by the findings of failure mode analysis, which showed that the majority of the samples of Group A (87.5%) showed a non-restorable fracture mode, denoting that the fracture toughness of composite resin is suboptimal, which could intensify the stresses at the crack-filler interface.

This finding is in agreement with Forster et al. [41], who suggested that direct composite restorations alone are not the ideal option for reinforcing or restoring root canal-treated teeth with MOD cavities. Nevertheless, the application and layering technique of resin-based composites influence the internal stress of direct restorations. Different layering approaches have been proposed to reduce internal stress, thus influencing mechanical behavior [42].

However, when the composite resin is combined with any fiber formulation (Glass fiber post, SFRC, LWUHMWPE), the fracture resistance increased significantly.

Glass fiber post significantly increased fracture resistance, as seen in Group B. This could be attributed to the fact that the modulus of elasticity of the glass fiber post is similar to dentin; therefore, the fiber post, when subjected to a compressive load, can better distribute the forces along the long axis of the post, decreasing the possibility of root fracture [43]. This finding is in contrast to the findings of Costa et al. [44], who found that the fracture resistance of premolars restored with composite resin and glass fiber post was not significant from that of premolars teeth restored with composite resin alone. Such contrary results could be attributed to the difference in cavity design that involved palatal cusp reduction in combination with the MOD cavity and the use of an adhesive resin cement for postcementation rather than the self-adhesive cement used in this study.

However, the use of SFRC with the composite resin (Group C) significantly increased fracture resistance significantly as compared to Group A, where the composite resin was used alone. This could be attributed to the toughening ability of SFRC, which contains short millimeter-scale randomly orientated E-glass fibers with a unique semi-interpenetrating polymer network structure that act as a crack stopper. This finding is in agreement with Sáry et al. [45], who found that bulk-applied SFRC covered with composite resin can reinforce MOD cavities in nonroot-filled molars.

Despite the statistically nonsignificant difference between Group B and Group C, the placement of a glass fiber post resulted in a nonrestorable fracture mode in the majority of samples (62.5%), while when SFRC was used, the majority of the fractures were restorable (75%). This might be related to the additional tooth structure removed during the preparation of the post and the possibility of microcracks initiation that may predispose to fracture [46]. Additionally, the use of a fiber post is a multistep approach that is more complex and sensitive, while SFRCs are easy to use and provide a time-efficient option for replacing dentin.

On the other hand, the results of this study showed that Group D and Group E recorded the highest mean values of fracture resistance, in which ribbond fibers were used in combination with composite resin, with a statistically nonsignificant difference between them. This could be attributed to the locked stitch interwoven framework structure of LWUHMWPE ribbon with nodal intersections that allow intrinsic stress and energy-absorbing mechanism [47]. The structure of polyethylene fibers improves the adaptation to the contours of the tooth [47]. The polyethylene ribbond fiber increases the load-bearing capacity of restorative material and inhibits crack propagation [48]. Moreover, the use of cold gas plasma manufacture of this material increases the adhesion to composite resins [49]. It is believed that the ribbond fiber inserted in the flowable resin used under composite restoration increases the fracture strength and microtensile bond strength to dentin [50]. The integrity of the restoration is maintained by the density of the fixed nodal intersections of ribbond fibers, which also transfers the stresses effectively through the restoration along well-defined paths [51].

When the ribbond was applied to the floor of the cavity (Group D), it was supposed to act as a crack stopper by changing the direction of stress and thus dissipating the strain allowing a stress-modifying effect to be formed, which in turn increases the overall toughness of the restoration [34, 52, 53]. This is in agreement with Eskitaşcıoğlu et al. [17], who suggested the combination of polyethylene fibers with a flowable composite resin to act as a stress absorber due to its lower modulus of elasticity. However, this finding disagrees with Akman et al. [38], who found that polyethylene fiber-reinforced restorations did not have a positive effect on fracture resistance but reduced cusp movement under loading. This might be attributed to the various experimental condition used to measure the fracture load. They used a relatively cross-head head speed (5 mm/min^−1^), as compared to a cross-head speed of (0.5 mm/min^−1^) used in most of the studies [9, 41, 54, 55].

On the other hand, the use of ribbond fibers in wallpapering technique (Group E) provided the highest fracture resistance mean value. This could be attributed to the fact that when the ribbond fibers placed against the cavity walls act similarly to the dentino-enamel complex, allowing tooth structure and composite materials to function in strain harmony [12]. Additionally, by adapting and polymerizing the ribbond fibers as closely as possible to the contours of the remaining tooth structure would allow a significant reduction in the volume of composite resin between tooth structure and the ribbond fiber, which in turn results in the protection of the remaining walls from both the stress from both the occlusal load and polymerization shrinkage [12].

An interesting advantage of using ribbond fibers is that the majority of the samples of Group D and Group E showed a restorable mode of fracture. The effectiveness of polyethylene fibers doesn't depend on the position of fibers within the cavity as there was no difference in the fracture resistance of Group D and Group E statically. This could be due to the use of ribbond fibers.

Whether on the cavity floor or against the walls, connecting the opposite walls and the fibers are not stretched or under any tension [45].

However, this study has several limitations. This study was performed *in vitro*. Although *in vitro* conditions provide standardized conditions, they may still not correlate to *in vivo* conditions. However, the single load-to-failure test is considered the cornerstone for evaluating materials in the initial stage. Furthermore, future studies shall include cyclic fatigue testing to better simulate clinical scenarios [56].

## 5. Conclusion

Incorporating ribbond fibers within the composite restoration, whether as a wallpapering or on the floor, significantly increased the fracture resistance of ETT.

## Figures and Tables

**Table 1:** Descriptive statistics of fracture resistance of the different groups.

Groups	*N*	Mean (*N*)	Std. deviation
A	8	689.625	98.3027
B	8	1270.38	260.936
C	8	1,409.875	225.85548
D	8	2025.5	161.29388
E	8	2,139.375	166.00511

**Table 2:** One-way ANOVA test for comparison of fracture resistance among the different groups.

ANOVA	Sum of squares	*df*	Mean square	*F*	Sig.
Between groups	11,218,135.400	4	2,804,533.850	76.906	0.000
Within groups	1,276,346.500	35	36,467.043		
Total	1,2494,481.900	39			

**Table 3:** Bonferroni test for multiple comparisons of fracture resistance between groups.

(*I*) Name of the groups	(*J*) Name of the groups	Mean difference (*I* ‒ *J*)	Std. error	Sig.
A	B	−580.75000	0.1074068	0.000
	C	−720.25000	0.1074068	0.000
	D	−1,335.87500	0.1074068	0.000
	E	−1,449.75000	0.1074068	0.000
B	C	−0.0846250	0.1074068	1.000
	D	−755.12500	0.1074068	0.000
	E	−869.00000	0.1074068	0.000
C	D	−615.62500	0.1074068	0.000
	E	−729.50000	0.1074068	0.000
D	E	−0.1138750	0.1074068	1.000

**Table 4:** Mode of fracture of the different groups.

Groups	Non-restorable fracture		Restorable fracture	
	No.	%	No.	%
A	7	87.5	1	12.5
B	5	62.5	3	37.5
C	2	25	6	75
D	3	37.5	5	52.5
E	2	25	6	75

## Data Availability

The data used to support the results of this study are contained in the article and can be obtained from the author at a reasonable request.
